# Smoking status and survival: impact on mortality of continuing to smoke one year after the angiographic diagnosis of coronary artery disease, a prospective cohort study

**DOI:** 10.1186/1471-2261-14-133

**Published:** 2014-10-01

**Authors:** Fadi Hammal, Justin A Ezekowitz, Colleen M Norris, T Cameron Wild, Barry A Finegan, for the APPROACH Investigators

**Affiliations:** Department of Anesthesiology & Pain Medicine, University of Alberta, 8-120 Clinical Sciences Building, Edmonton, Alberta T6G2G3 Canada; Mazankowski Alberta Heart Institute, Edmonton, Alberta Canada; Faculty of Nursing, University of Alberta, Edmonton, Alberta Canada; School of Public Health, University of Alberta, Edmonton, Alberta Canada; Division of Cardiovascular Surgery, University of Alberta, Edmonton, Alberta Canada; Division of Cardiology, University of Alberta, Edmonton, Alberta Canada

**Keywords:** Smoking cessation, Coronary artery disease, Coronary artery bypass grafting, Percutaneous coronary intervention

## Abstract

**Background:**

Smoking is an undertreated risk factor for coronary artery disease (CAD) and is associated with adverse outcomes after myocardial infarction. Aims of our study were to determine if management of CAD by medical therapy (MT) alone or with coronary artery bypass grafting (CABG) or percutaneous coronary intervention (PCI) influence smoking status at one year following angiography and if a change in smoking status at one year influences long term survival.

**Methods:**

Prospective cohort study using the APPROACH registry. Two cohorts were examined: (1) 11,334 patients who returned a one year follow-up questionnaire; (2) 4,246 patients propensity-matched based on their post-angiography treatment - MT or revascularization (RV). Multivariate modeling and survival analysis were used.

**Results:**

In the propensity-matched cohort, quit rates at one year were greater among CABG patients (68%) than PCI (37%) or MT patients (47%). Smokers in the RV group, who self-reported quitting at one year, had a significantly reduced mortality compared to those who continued to smoke.

**Conclusions:**

CABG patients were more likely to quit smoking than those treated with MT alone or PCI. Quitting smoking was associated with improved long-term survival; smoking remains a key risk factor for mortality in patients with CAD. These data underscore the importance of nicotine addiction management in patients with CAD and the need to emphasize cessation particularly in those patients undergoing MT or PCI.

## Background

Cigarette smoking is associated with the development of coronary artery disease (CAD) [1] and influences short and long term outcomes of patients who smoke after diagnosis [2–4]. Smoking cessation substantially reduces the risk of total coronary heart disease mortality [5, 6], and can reduce the need for revascularization procedures [7]. The single most common CAD risk factor among patients hospitalized for initial myocardial infarction is hypertension (44%), followed by smoking (23%) [8]. Globally, smoking is still considered to be responsible for up to one third of the mortality associated with cardiovascular disease [9]. Despite the availability of smoking cessation therapies and the publication of excellent guidelines outlining the measures required to enhance the quit rates of smokers with CAD [10], there continues to be poor uptake by clinicians and patients of the behavioural and pharmacological measures available to assist patients with established CAD to quit smoking [11].

The objectives of the current study were to assess 1) if different management strategies: medical therapy alone (MT), MT and coronary artery bypass grafting (CABG) or MT and percutaneous coronary intervention (PCI) influence quit rates at one year following diagnostic angiography, and 2) if quitting smoking at one year influences long term survival independent of the treatment strategy.

## Methods

### Design and setting

The Alberta Provincial Project for Outcomes Assessment in Coronary Heart disease (APPROACH) registry captures data on all patients who undergo coronary angiography in Alberta, Canada. This study reports on data collected between January 2003 and March 2010. Database and data collection methods have been previously described in detail [12]. In brief, data collected includes socio-demographic characteristics, smoking status, clinical co-morbidities, prior cardiac events, indication for angiography (which includes detail regarding a clinical event if present), ejection fraction, coronary anatomy and all treatments received (MT, CABG, PCI) following angiography. Clinical indications for angiography included acute/recent MI, stable angina, unstable angina, and other. Data in APPROACH are linked quarterly with the Service Alberta Ministry–Vital Statistics to capture mortality and date of death (99.9% follow up).

All patients in APPROACH who consented to follow-up during the study period were sent a questionnaire one year after their index angiography. Two questions related to tobacco: *Do you currently smoke cigarettes?* and *If you quit, how long ago did you quit?*

The APPROACH study and sub-studies are approved by the ethical review boards at the University of Alberta and the University of Calgary.

### Statistical analysis

Pearson Chi-Squared test for categorical variables and t-test for continuous variables were used to compare baseline clinical characteristics across strata. Patients were initially stratified based on their smoking status at time of angiography into current smokers and non-smokers. Patients were subsequently stratified into four categories based on their smoking status at angiography and one year: 1) patients who reported being smokers at baseline and at follow-up, 2) patients who reported being smokers at baseline and non-smokers at follow-up, 3) patients who reported being non-smokers at baseline and at follow-up, and 4) patients who reported being non-smokers at baseline and smokers at follow-up. The latter constitute a small number of patients (n = 64), and were excluded from further analysis. A Pearson Chi-Squared test was used to assess the association between the treatment that the patient received after angiography and smoking status at one year follow-up.

Two cohorts were examined: Cohort *A*, the entire group who returned an analysable questionnaire at one year, and, Cohort *B*, a group from Cohort *A,* matched based on the propensity to undergo revascularization. The propensity score was calculated as the probability of undergoing revascularization (PCI or CABG) on the observed baseline (measured at recruitment) characteristics. This technique allows for a high number of confounding variables and has been used to create a stratum of subjects who can be matched on the propensity score whereby exposure is not confounded with measured baseline covariates. The propensity score was calculated using logistic regression. The following variables were included in the model: age, sex, smoking status, pulmonary disease, cerebrovascular disease, renal disease, heart failure, diabetes, dialysis, hypertension, hyperlipidemia, liver/GI disease, malignancy, peripheral vascular disease, prior MI, prior PCI, prior CABG, prior lytic therapy, the indication for angiography including MI, stable angina, unstable angina or other, the coronary anatomy and the ejection fraction. Greedy matching techniques were applied to match patients who were revascularized to patients who were treated with medical management by matching the participants with the nearest propensity score, i.e. within 3 decimal places of the propensity score for each case. Overlap of propensity scores between revascularized and medically managed patients were evaluated using histograms, Chi-Squared values and probability values. Differences in baseline factors between groups were calculated before and after propensity adjustment to assess balance.

Survival analysis was conducted; survival tables and log rank tests were used to determine if there was a statistically significant survival difference between revascularized and medically managed patients.

*Sensitivity analysis* was conducted in order to account for the effect of disease progress and medical management changes over time by splitting the follow up period into two 45 month periods.

All analyses were conducted using SPSS version 19.0 (IBM SPSS, Armonk, NY).

## Results

In all 29,230 patients were invited to complete the APPROACH survey at one year after their procedure. The response rate to the survey was 57.4% with 38.7% returning a fully completed questionnaire that was suitable for analysis. Surveys were not sent to the families of subjects who had died within one year (n = 1968; 6.7% of total patient population), 33% of those who died were smokers at time of angiography. Data on the latter group consisted of demographic and other information collected at the time of angiography and mortality data obtained from Service Alberta Ministry – Vital Statistics.

**Figure 1:**
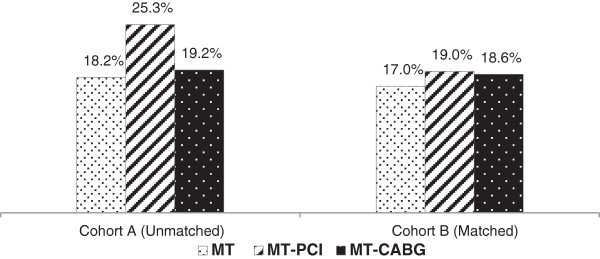
**Smoking status at the time of angiography.** MT: Medial Treatment, PCI: Percutaneous Coronary Intervention, CABG: Coronary Artery Bypass Grafting.

**Figure 2:**
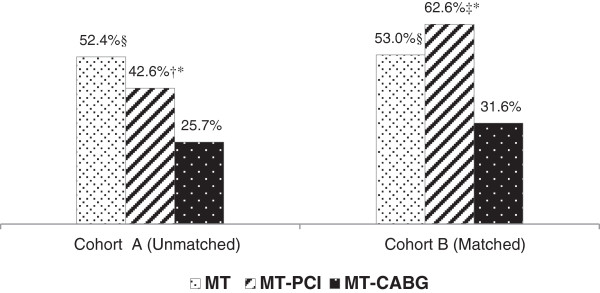
**Smoker who kept smoking at one year after angiography.** MT: Medial Treatment, PCI: Percutaneous Coronary Intervention, CABG: Coronary Artery Bypass Grafting. †p value PCI vs. MT <0.001; *p value PCI vs. CABG <0.001; §p value MT vs. CABG <0.001; ‡p value PCI vs. MT = 0.007.

The smoking status of at baseline and at one year for both Cohorts is shown in (Figures 1 and 2). In Cohort *A*, CABG patients were more likely to report quitting at follow up. In Cohort *B*, groups were well matched with respect to smoking status at time of angiography and as in Cohort *A*, CABG patients were more likely to quit.

In Cohort *A*, patients who smoked were significantly younger at time of index angiography, were more likely to present with acute coronary syndrome, to had a prior MI and to have required an emergency or urgent in-hospital angiography (Table 1). These differences remained significant when patients were stratified according to post-angiography treatment strategy (Table 2). Additionally, characteristics of smokers who quit compared with those who did not quit in *Cohort A* are shown in Table 3.

**Table 1:** **Baseline characteristics stratified by smoking status at angiography (Cohort** ***A*** **, unmatched)**

Clinical characteristics	Non-smoker	Smoker	p-value
No. (%)	8750 (77.2)	2584 (22.8)	
Age, Mean (SD)	66.4 (10.4)	58.7 (9.8)	<0.001
Female (%)	21.8	22.4	
Diabetes Mellitus (%)	22.9	19.6	<0.001
Hyperlipidemia (%)	78.5	79.2	
Pulmonary disease (%)	12.6	14.0	
Heart Failure (%)	8.7	9.1	
Hypertension (%)	70.5	62.6	<0.001
CVD (%)	5.9	4.1	<0.001
PVD (%)	5.9	7.2	0.015
ST-Elevated MI (%)	21.5	38.2	<0.001
Non ST-Elevated MI (%)	21.9	29.3	<0.001
Prior MI (%)	22.4	24.9	0.008
Prior PCI (%)	4.8	3.3	0.001
Prior CABG (%)	3.7	1.4	<0.001
Prior lytic (%)	4.8	10.6	<0.001
ACS (%)	55.5	73.8	<0.001
Indication for angiography (%)			<0.001
Stable angina	37.7	20.7	
MI	38.9	60.0	
Unstable angina	18.6	15.1	
Other*	4.9	4.1	
Priority to angiography (%)			<0.001
Emergency	10.5	16.1	
Urgent in Hospital	47.2	59.0	
Urgent out of hospital	14.5	9.2	
Planned	27.1	14.4	
Unknown	0.8	1.3	
Ejection fraction (%)			<0.001
>50%	62.3	57.5	
35-50%	17.6	22.1	
20-34%	3.6	4.6	
<20%	0.8	1.2	
Not done	8.7	7.8	
Coronary Anatomy (%)			<0.001
Normal	0	0	
<50% disease	0.2	0.1	
Low risk (1–2 VD)	53.2	61.3	
High Risk (2–3 VD)	36.7	30.9	
Left Main disease	9.8	7.6	
First treatment after angiography (%)			<0.001
Medical	21.4	17.6	
CABG	22.8	18.4	
PCI	55.8	64.0	

SD: Standard deviation, BMI: body mass index, CVD: Cerebrovascular disease, PVD: Peripheral vascular disease, MI: myocardial infarction, PCI: Percutaneous Coronary Intervention, CABG: Coronary Artery Bypass Grafting, ACS: Acute coronary syndrome, VD: vessel disease. *Other include silence ischemia, evaluation of serious arrhythmia, preoperative assessment, research protocol angiography.

**Table 2:** **Baseline characteristics stratified by treatment (Cohort** ***A*** **)**

Clinical characteristics	Medical	Revascularization	p-value
No. (%)	2326 (20.5)	9008 (79.5)	
BMI, Mean (SD)	28.8 (5.6)	28.8 (5.1)	
Age, Mean (SD)	65.5 (10.8)	64.2 (10.7)	<0.001
Female (%)	24.9	21.1	<0.001
Current smoker (%)	19.6	23.6	<0.001
Diabetes Mellitus (%)	26.9	20.9	<0.001
Hyperlipidemia (%)	78.9	78.6	
Pulmonary disease (%)	16.3	12.1	<0.001
Heart Failure (%)	13.7	7.5	<0.001
Hypertension (%)	72.8	67.6	<0.001
CVD (%)	7.5	5.0	<0.001
PVD (%)	8.5	5.6	<0.001
ST-Elevated MI (%)	11.7	28.9	<0.001
Non ST-Elevated MI (%)	19.1	24.8	<0.001
Prior MI (%)	28.3	21.6	<0.001
Prior PCI (%)	5.7	4.1	0.001
Prior CABG (%)	7.2	2.1	<0.001
Prior lytic (%)	2.6	7.0	<0.001
ACS (%)	42.8	64.1	<0.001
Indication for angiography (%)			<0.001
Acute/recent MI	26.7	48.1	
Unstable angina	17.4	17.9	
Stable angina	48.1	30.1	
Other*	7.8	3.9	
Priority to angiography (%)			<0.001
Emergency	2.2	14.2	
Urgent in hospital	45.5	51.0	
Urgent out of hospital	16.8	12.3	
Planned	35.4	21.4	
Unknown	0.2	1.0	
Ejection fraction (%)			<0.001
>50%	62.0	61.0	
35-50%	16.0	19.3	
20-34%	6.0	3.2	
<20%	2.0	0.6	
Not done	13.9	15.9	
Coronary Anatomy (%)			<0.001
Normal	0.2	0.0	
<50% disease	0.3	0.1	
Low risk (1–2 VD)	65.5	52.4	
High Risk (2–3 VD)	28.6	37.1	
Left Main disease	5.4	10.3	

SD: Standard deviation, BMI: body mass index, CVD: Cerebrovascular disease, PVD: Peripheral vascular disease, MI: myocardial infarction, PCI: Percutaneous Coronary Intervention, CABG: Coronary Artery Bypass Grafting, ACS: Acute coronary syndrome, VD: vessel disease. *Other include silence ischemia, evaluation of serious arrhythmia, preoperative assessment, research protocol angiography.

**Table 3:** **Baseline characteristics by smoking status at one year after angiography (Cohort** ***A*** **)**

Clinical characteristics	Smoker at one year	Quitter at one year	p-value
No. (%)	1064 (41.2)	1519 (58.8)	
BMI, Mean (SD)	28.6 (5.6)	28.8 (5.2)	0.4
Age, Mean (SD)	58.4 (9.6)	58.9 (9.9)	0.3
Female (%)	27.3	19.0	<0.001
Diabetes Mellitus (%)	19.0	20.1	0.5
Hyperlipidemia (%)	79.7	78.9	0.6
Pulmonary disease (%)	15.3	13.1	0.1
Heart Failure (%)	8.4	9.6	0.3
Hypertension (%)	63.1	62.3	0.7
CVD (%)	4.4	3.9	0.6
PVD (%)	8.9	6.1	0.007
ST-Elevated MI (%)	34.7	40.6	0.002
Non ST-Elevated MI (%)	27.3	30.7	0.07
Prior MI (%)	26.2	23.9	0.2
Prior PCI (%)	3.8	3.0	0.3
Prior CABG (%)	1.3	1.4	0.9
Prior lytic (%)	8.6	12.0	0.006
ACS (%)	70.2	76.4	0.001
Indication for angiography (%)			<0.001
Acute/recent MI	55.0	63.5	
Unstable angina	16.1	14.5	
Stable angina	24.9	17.8	
Other*	4.0	4.2	
Priority to angiography (%)			0.01
Emergency	13.8	17.7	
Urgent in hospital	58.2	59.5	
Urgent out of hospital	10.1	8.6	
Planned	16.5	13.0	
Unknown	1.4	1.2	
Ejection fraction (%)			0.09
>50%	59.8	56.0	
35-50%	22.3	21.9	
20-34%	4.1	4.8	
<20%	0.9	1.4	
Not done	14.9	16.0	
Coronary Anatomy (%)			<0.001
Normal	0.0	0.1	
<50% disease	0.0	0.2	
Low risk (1–2 VD)	69.9	55.2	
High Risk (2–3 VD)	25.1	35.0	
Left Main disease	4.9	9.5	

SD: Standard deviation, BMI: body mass index, CVD: Cerebrovascular disease, PVD: Peripheral vascular disease, MI: myocardial infarction, PCI: Percutaneous Coronary Intervention, CABG: Coronary Artery Bypass Grafting, ACS: Acute coronary syndrome, VD: vessel disease. *Other include silence ischemia, evaluation of serious arrhythmia, preoperative assessment, research protocol angiography.

The baseline characteristics of Cohort *B* were remarkably well matched (Table 4). There were no significant differences between initial treatment strategies and subsequent cross-over to another treatment strategy - 86% and 87% of patients in MT and RV groups did not undergo any other treatment over the follow-up period (Table 5). Self-reported smoking *cessation rates* at one year following angiography were significantly higher among CABG patients (68%) than PCI (37%, p < 0.001) or MT patients (47%, p <0.001) (Figure 2, Table 6).

**Table 4:** **Baseline characteristics stratified by treatment (Cohort** ***B*** **)**

Clinical characteristics	Medical	Revascularization	p-value
No.	2123	2123	
BMI, Mean (SD)	28.8 (5.6)	28.8 (5.2)	1
Age, Mean (SD)	66.5 (10.8)	66 (10.3)	0.2
Female (%)	24.6	25.4	0.6
Current smoker (%)	18.6	18.5	1
Renal disease (%)	3.9	3.9	1
Liver/ GI disease (%)	8.2	8.9	0.4
Diabetes Mellitus (%)	26.6	25.6	0.4
Hyperlipidemia (%)	78.8	78.9	1
Malignancies (%)	5.1	5.3	0.7
Pulmonary disease (%)	16.9	17.2	0.8
Heart Failure (%)	13.8	12.0	0.07
Hypertension (%)	72.4	72.5	1
CVD (%)	7.6	8.0	0.7
PVD (%)	8.6	8.7	0.9
ST-Elevated MI (%)	11.4	10.7	0.5
Non ST-Elevated MI (%)	18.8	18.6	0.8
Prior MI (%)	28.0	27.5	0.7
Prior PCI (%)	5.9	6.2	0.6
Prior CABG (%)	7.2	5.9	0.1
Prior lytic (%)	2.7	2.7	1
ACS (%)	42.1	41.9	0.9
Indication for angiography (%)			0.9
Acute/recent MI	26.1	25.5	
Unstable angina	17.1	17.6	
Stable angina	49.3	49.0	
Other*	7.5	7.9	
Ejection fraction (%)			0.1
>50%	63.0	66.0	
35-50%	16.0	14.6	
20-34%	6.2	5.1	
<20%	1.8	1.3	
Not done	7.0	6.2	
Coronary Anatomy (%)			0.7
Normal	0.2	0	
<50% disease	0.3	0.3	
Low risk (1–2 VD)	66.1	67.2	
High Risk (2–3 VD)	28.3	27.5	
Left Main disease	5.1	4.9	

SD: Standard deviation, BMI: body mass index, CVD: Cerebrovascular disease, PVD: Peripheral vascular disease, MI: myocardial infarction, PCI: Percutaneous Coronary Intervention, CABG: Coronary Artery Bypass Grafting, ACS: Acute coronary syndrome, VD: vessel disease. *Other include silence ischemia, evaluation of serious arrhythmia, preoperative assessment, research protocol angiography.

**Table 5:** **Treatments received after first treatment during follow-up period (Cohort** ***B*** **)**

		First treatment after catheterization					
		Medical		CABG		PCI	
		N	%	N	%	N	%
**Secondary treatment**	None	1818	85.6	487	87.1	1359	86.9
	PCI	95	4.5	39	7.0	59	3.8
	CABG	124	5.8	21	3.8	73	4.7
	Lytic	56	2.6	3	0.5	47	3.0
	PCI & Lytic	1	0.0	0	0.0	3	0.2
	CABG & Lytic	0	0.0	0	0.0	1	0.1
	PCI & CABG	28	1.3	7	1.3	21	1.3
	PCI & CABG & Lytic	1	0	2	0.4	1	0.1
Total		2123	100.0	559	100.0	1564	100.0

PCI: Percutaneous Coronary Intervention, CABG: Coronary Artery Bypass Grafting.

**Table 6:** **Treatment association with self-reported smoking status at 1 year post angiography**

Smoking status at	Treatment			χ ^2^(p-value)		
	MT no. (%)	CABG no. (%)	PCI no. (%)	MT vs. CABG	MT vs. PCI	CABG vs. PCI
Cohort *A* smokers quit at one year	217 (47.6)	353 (74.3)	949 (57.4)	69.9 (<0.001)	14.0 (<0.001)	44.2 (<0.001)
Cohort *B* smokers quit at one year	185 (47.0)	65 (68.4)	111 (37.4)	14.11 (<0.001)	6.35 (0.007)	28.05 (<0.001)

PCI: Percutaneous Coronary Intervention, CABG: Coronary Artery Bypass Grafting.

### Survival analysis

Patients were followed for a mean of 42.2 months (SD 24.9 months). Non-smokers had a significantly higher long term survival rate compared with smokers in the RV group (93% vs. 89%, p <0.05), but not in those in MT (89% vs. 88%, p = 0.8) (Table 7). Smokers at baseline who had quit at one year had greater long term survival rate compared with those who continued to smoke after revascularization (95% vs. 89%, p < 0.05), a trend towards reduced mortality was also observed in patients in the MT group (93% vs. 88%, p = 0.2) (Table 7).

**Table 7:** **Survival analysis**

Cohort ***A***(Unmatched)					
	Smoking status	Total N	N of events	Censored	
				N	Percent
	Smokers	1064	85	979	92.0%
	Smokers who quit at one year	1519	65	1454	95.7%
	Non-smokers	8606	610	7996	92.9%
**Cohort** ***B*** **(matched)**					
**Treatment**	**Smoking status**	**Total N**	**N of events**	**Censored**	
				**N**	**Percent**
MT	Smokers	209	25	184	88.0%
	Smokers who quit at one year	185	13	172	93.0%
	Non-smokers	1697	184	1513	89.2%
RV	Smokers*^§^	216	24	192	88.9%
	Smokers who quit at one year	176	9	167	94.9%
	Non-smokers	1699	123	1576	92.8%

*Log rank significance compared to smokers who quit at one year < 0.05.

^§^Log rank significance compared to non-smokers < 0.05.

MT: Medical Treatment; RV: Revascularization.

Our sensitivity analysis showed no alteration in the smoking-related survival pattern, however, survival improved for all patients regardless of the index treatment strategy in the second follow-up period (MT: 85.7% vs. 93.1%; CABG: 84.5% vs. 91.2%; and PCI: 91.4 vs. 96.2%, first vs. second period, respectively).

## Discussion

The overwhelming impact of tobacco smoking on the time course of the appearance of CAD is illustrated in our study, confirming the findings of the National Registry of Myocardial Infarction 2 study [13]. We found that smokers were more likely to present on an urgent basis and suffer an STEMI relative to non-smokers. Despite successful efforts to promote a standardized approach to the management of patients following acute myocardial infarction (AMI), the prevalence of smoking in this population group has remained largely unchanged over the last decade [14].

A key treatment goal following the diagnosis of CAD is to initiate effective secondary prevention measures, including nicotine addiction management for those patients who smoke [10]. Our data point to the importance of rehabilitation care for smokers following diagnostic or any procedural interventions, particularly those who undergo PCI. In Alberta, patients who have been diagnosed with CAD through angiography are referred to outpatient cardiac risk management programs. Unfortunately, data on referral rates by clinicians and uptake of these programs by patients were unavailable, nevertheless, evidence from other sources indicate that referral to out-patient cardiac rehabilitation services remains low [15] especially in the socially disadvantaged [16]. Recent Medicare data indicate that those who have undergone CABG are more than twice as likely as others to use out-patient cardiac rehabilitation services [16]. Parashar *et al.* [17] tracked participation rates of patients after an AMI enrolled into cardiac rehabilitation programs after discharge from hospital and found that current smokers, those with lower socioeconomic status and those who had undergone PCI were the least likely to participate in the programs.

To our knowledge, this is the largest study to assess the impact of smoking and smoking cessation on outcomes in a broad and generalizable propensity matched cohort. We found a strong association with a lower survival in patients who smoked who had undergone revascularization, even after adjusting for key variables and performing a propensity matching to account for variations in patient and clinician decisions on further care.

For smokers with CAD, quitting smoking is the most effective secondary preventive measure in reducing mortality [18, 19]. The evidence that quitting smoking is associated with a survival benefit relative to continuing to smoke is overwhelming. These data, first elegantly described in a prospective cohort study by Richard Doll in the British Doctors Study [20], have been recently re-enforced by the Nurses’ Health Study conducted in over 100,000 women between 1982 and 2004 [21]. In a systematic review of twenty observational non-randomized studies, Critchley *et al.* [22] found that quitting smoking was associated with substantial risk reduction (36%) of all-cause mortality among patients with CAD. The risk reduction for other secondary prevention measures was 29% for statins [23], 15% for Aspirin [24], 23% for β-blockers [25], and 23% for angiotensin converting enzyme inhibitors [26]. An earlier systematic review found comparable risk reduction [27].

Among patients with CAD, quitting smoking reduces the risk of recurrent events, improves patients’ quality of life and is cost-effective [28, 29]. Compared with other secondary preventive measures, smoking cessation is both an effective and cost-effective intervention [18, 22]. Our data provide an impetus to health systems, policy makers and patients to ensure this remains a focus of care regardless of the CAD management strategy. The Canadian Cardiovascular Society has emphasised the priority of identifying and documenting smoking status and providing smoking cessation support for all smokers admitted to hospital with CAD [10]. The outcome of implementing such programs highly depends on the program structure and duration. Short inpatient education programs have not increased smoking cessation rates at one year and at five years [30]. More intensive behavioral interventions with at least one month of supportive contacts after hospital discharge increased cessation rates [31, 32], as does add pharmacological management [30, 33].

We observed lower mortality rates across intervention groups when data were analysed into two successive 45-month periods. These findings could be a reflection of an improvement in CAD management strategies. Despite this, we found long-term survival rates were consistently lower among patients with CAD who smoked compared to non-smokers or smokers who quit smoking. The observed reduction in mortality is similar to the results reported in the Coronary Artery Surgery Study after 10 years of follow-up [34], where patients who continued to smoke had significantly lower survival rates compared with quitters in CABG group (84% vs. 68%, p = 0.018) and not a significant difference for patients in the medical treatment arm (75% vs. 71%).

Observations that smokers had decreased mortality rates following acute myocardial infarction, in the era when fibrinolysis was the leading reperfusion strategy, have introduced the term “smokers’ paradox” to the medical literature. However, our findings, as well other recent studies do not support this observation [35]. Likewise, a recent systematic review reported that all contemporary studies that follow-up with patients for longer than one year do not have evidence for such paradox attributing the existence of this phenomenon to the difference in the baseline risk and comorbidities, to the difference in the pathogenesis of acute coronary events between smokers and non-smokers, or to methodological errors in studies [36]. Our data lend further evidence that quitting smoking, particularly if revascularization has been performed, is beneficial.

We have shown in our study, albeit in a relatively small sample size, a trend of greater long term survival rate for smokers who self-reported quitting at one year compared with non-smokers. The mechanisms underlying this effect were not the focus of this study. However, the deleterious consequences of smoking on stress hormone release, blood pressure, thrombotic tendency and vascular biology have been well described and aptly summarized in a recent review by *Messner and Bernard* [37].

### Strengths and limitations

This study used data from an observational registry and has limitations that generally apply to observational analyses. The APPROACH registry captures all patients for the catchment area in Alberta (population 3.5 million) and has complete data on all patients undergoing angiography in the province and their subsequent treatment and outcome. Hence, it is highly generalizable and reflects clinical care but may not be able to capture all variables that influence survival or would eliminate or enhance any of our statistically significant results. Variables usually absent from larger observational datasets including number of diseased coronary arteries, EF and coronary anatomy are available enhancing the ability to better describe the population being studied. Propensity-based matching attempts to eliminate other bias associated with conventional multivariable modeling and treatment selection. This study used self-reported smoking cessation rates to assess smoking status and survey methodology. Although biochemical validation of smoking status is considered a more robust method in assessing cessation, self-reported cessation rates are still a reliable method in assessing smoking status in patients admitted to hospital [38]. Unfortunately, we do not know the duration of smoking history or life time exposure to tobacco of our population; these data were not included in the Approach database.

## Conclusions

Our data indicate that smokers who have undergone CABG were more likely to report smoking cessation at one year following the angiography than those who are treated with PCI or MT. Quitting smoking is associated with improved long-term survival, particularly among revascularized patients. Smoking remains an undertreated risk factor among patients with angiographically proven CAD. Quitting smoking should be a key objective of management in patients with CAD.
